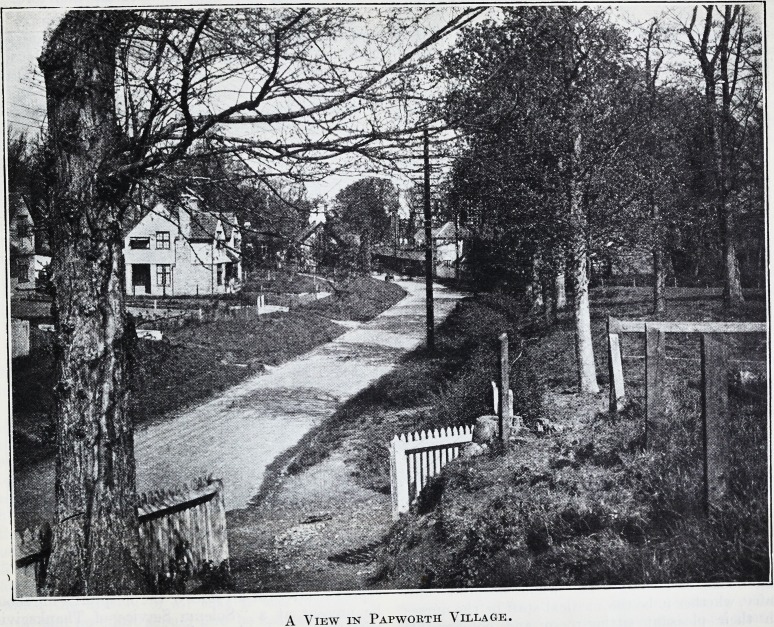# Papworth Village Settlement

**Published:** 1924-08

**Authors:** 


### PAPWORTH VILLAGE SETTLEMENT.

### THE CONSUMPTIVE AS WAGE EARNER.

The visit of the Duke and Duchess of York to Papworth, in Cambridgeshire, to open new cottages is a reminder of the interest of the Royal Family in the remarkable experiment for which the word "Papworth" stands. It is something more. It is a definite indication of the progress which is being made. Indeed, says Dr. P. C. Varrier-Jones, the Medical Director, if we did not make progress we should very soon lose sight of the ideal which we set out to attain; if we did not go forward, we should lose ground and deterioration would set in. The immediate occasion of the Royal visit was the formal opening of one of six cottages that are nearing completion, this being the first instalment of twenty-five new cottages that are being built from a Government grant of £10,000. This grant was made after Mr. Neville Chamberlain, then Minister of Health, visited the Colony in 1923.

Polishing Shop and the Cabinet-making Department.

The Royal visitors were received at the hall by Sir Clifford Allbutt, president of the colony, Mrs. Marcus Dimsdale, hon. secretary, and Dr. Varrier-Jones. A tour was made of the workshops, the Duke and Duchess showing particular interest in some new machinery installed in the leather work-shops, where bags of all kinds are made. Their Royal Highnesses accepted a lady's dressing-case and a gentleman's suit-case made in the department. A short drive was then taken through the village, and upon their return to the hall they stopped to open the new cottage. Mr. Dunn, the architect, handed to the Duke a golden key to unlock the front door. The visitors then went over the cottage, which had been tastefully furnished with plenishings made in the colony workshops. They both expressed themselves as being delighted with the cosy little home. They next visited some of the wards in the hospital and chatted with several of the patients. Altogether their Royal Highnesses spent nearly an hour and a half at the colony and departed amid the cheers of the crowd.

It is no longer possible to speak of the creation of a village occupied by consumptives and their families as a dream. Here, at Papworth, is a village community of 200 inhabitants (including seventy children). The new cottages springing up indicate that the community is not only a going but a go-ahead concern. In the picturesque cottages the

Industries Stores.

married men live with their families. That ensures the first vital consideration for success—family life. In the hostels for men and the recently completed hostel for women, the single are comfortably and socially housed. All the workshops are busy with the activities of men and women who realise by the handling of their earnings, and by the positions of responsibility and trust to which they may and do attain, that they are responsible wage-earning members of the community. That is the second vital consideration.

The occupations comprise carpentry and joinery, cabinet-making, portmanteau-making, boot-making and repairing, horticulture, pig and poultry farming, printing, bee keeping, game keeping, hand-made jewellery, etc. There are no highly paid fit men acting as officials in the industries; all such posts are held by ex-patients who prove to be qualified for them. This, says Dr. Varrier-Jones, is one of the secrets of the success of the Colony—encouragement in a practical form of that abundant hope which is so characteristic of the consumptive. The social needs of this community are provided for by sports clubs, recreation rooms, cinematograph theatre, etc., and the parish church and Nonconformist chapel supply the spiritual wants. These things, together with a hospital, make up the first village settlement for the consumptive.

But let there be no mistake. If other enthusiasts are thinking of starting village settlements on these lines, let them first discover their Mrs. Marcus Dimsdale and their Dr. Varrier-Jones. The Committee of the Colony in presenting their Annual Report showing transactions on their capital account approaching £140,000 and a turnover of £23,000 a year on their income and expenditure account, are without doubt right in attributing their funds and their development largely to Mrs. Dimsdale's initiative and organisation. Of the Medical Director they say:—"Dr. Varrier-Jones who never allows the work to stand still, is untiring in the energy and originality that he displays in the interests of the Colony, and to him and his able assistant staff the Committee are again most grateful." He is, in fact, family doctor, consultant, father of the family, business superintendent, guide, philosopher, and friend, all rolled into one.

A View in Papworth Village.

### VOLUNTARY AID IN LIVERPOOL.

The Fourteenth Annual Report of the Liverpool Council of Voluntary Aid records another year of quiet growth. The Council was founded in 1909 to promote and assist charitable work in the city and its neighbourhood, and is obviously of great value to voluntary social effort. Every year the Council helps the Liverpool Charities to an increasing extent with both money and services.

## Figures and Tables

**Figure f1:**
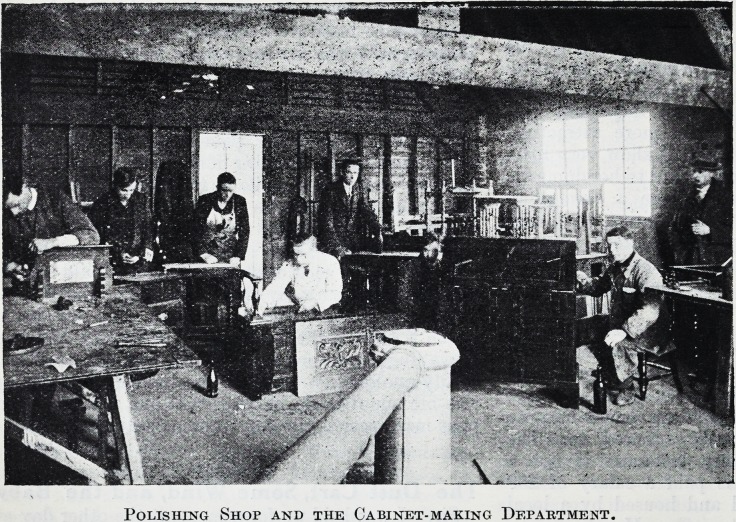


**Figure f2:**
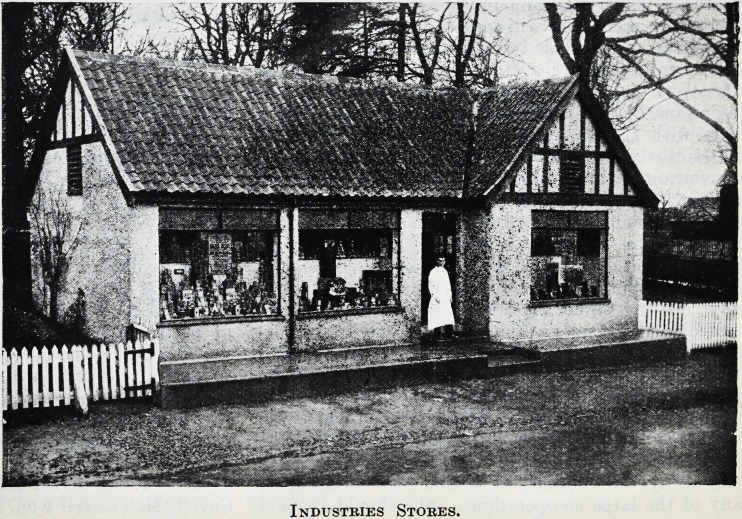


**Figure f3:**